# Prediction of the structural interface between fibroblast growth factor23 and Burosumab using alanine scanning and molecular docking

**DOI:** 10.1038/s41598-022-18580-3

**Published:** 2022-08-30

**Authors:** Karnpob Kanhasut, Kannan Tharakaraman, Mathuros Ruchirawat, Jutamaad Satayavivad, Mayuree Fuangthong, Ram Sasisekharan

**Affiliations:** 1 Program in Applied Biological Sciences, Chulabhorn Graduate Institute, 54 Kamphaeng Phet 6 Rd, Bangkok, 10210 Thailand; 2 Koch Institute for Integrative Cancer Research, Massachusetts Institute of Technology, Cambridge, MA 02139 USA; 3 Translational Research Unit, Chulabhorn Research Institute, Bangkok, 10210 Thailand; 4 Center of Excellence on Environmental Health and Toxicology (EHT), OPS, MHESI, Bangkok, Thailand; 5 Laboratory of Pharmacology, Chulabhorn Research Institute, 54 Kamphaeng Phet 6 Rd, Bangkok, 10210 Thailand; 6 Program in Environmental Toxicology, Chulabhorn Graduate Institute, 54 Kamphaeng Phet 6 Rd, Bangkok, 10210 Thailand; 7 Department of Biological Engineering, Massachusetts Institute of Technology, Cambridge, MA 02139 USA

**Keywords:** Protein design, Protein structure predictions, Molecular modelling, Endocrine system and metabolic diseases, Biological therapy

## Abstract

Burosumab, an FGF23 targeting monoclonal antibody, was approved by the FDA in 2018 for use in children and adults with X-linked hypophosphatemia (or XLH). While several clinical studies have demonstrated the long-term safety and efficacy of Burosumab, the molecular basis of FGF23-Burosumab interaction which underpins its mechanism of action remains unknown. In this study, we employed molecular docking combined with alanine scanning of epitope and paratope to predict a model of FGF23-Burosumab interaction. Then, we used the model to understand the species-species cross-reactivity of Burosumab and to reverse engineer mouse FGF23 with 'back to human' mutations to bind Burosumab. Finally, we redesigned the CDRs with two mutations to engineer an affinity enhanced variant of the antibody. Our study provides insights into the FGF23-Burosumab interaction and demonstrates that alanine-scanning coupled with molecular docking can be used to optimize antibody candidates (e.g., structure-guided affinity maturation) for therapeutic use.

## Introduction

FGF23 is a circulating growth factor secreted by osteocytes that is essential for phosphate homeostasis. In kidney proximal tubular cells, FGF23 inhibits phosphate reabsorption and leads to decreased synthesis and enhanced catabolism of 1,25-dihydroxyvitamin D3 (1,25[OH]2 D3). Excess levels of FGF23 cause renal phosphate wasting and suppression of circulating 1,25(OH)2 D3 levels and are associated with several hereditary hypophosphatemic disorders with skeletal abnormalities, including X-linked hypophosphatemic rickets (XLH) and autosomal recessive hypophosphatemic rickets (ARHR). Therefore, targeted inhibition of FGF23 presents an attractive opportunity to ameliorate XLH and other bone disorders.

Like other FGF family members, FGF23 possesses a conserved N-terminal region having a β-trefoil structure for binding to its fibroblast growth factor receptor (FGFR1). Unlike other paracrine FGFs, FGF23 binds weakly to the D2 and D3 domains of FGFRs. Binding of FGF23 and α-klotho, a cofactor that binds to the C-terminal region of FGF23, to FGFR1 activates the signaling cascade^1^. Specifically, α-klotho acts as a bridge by bringing FGF23 and FGFR splice c isoform (FGFR1c) in close proximity enabling the formation of the ternary complex (Supplementary Fig. S1).

Burosumab (Crysvita) is an anti-FGF23 neutralizing antibody that was approved by FDA for use in children and adults with XLH. Burosumab targets the N-terminal region of FGF23, blocking the interaction with the FGFR1. The antibody inhibits FGF23 signaling of human, monkey and rabbit FGF23, but not mouse or rat FGF23^2^. The antibody has been examined for its safety and efficiency through several clinical studies in both adult and pediatric patients^3–5^. Recently, Burosumab has been approved for tumor-induced osteomalacia (TIO)^6^, while its implication in other hypophosphatemia-related conditions is being explored^7^. While growing number of studies point to Burosumab’s excellent safety and efficacy profile, the molecular basis of its interaction with FGF23 leading to its mechanism of action remains unsolved.

X-ray crystallography is generally an approach of choice for solving antigen–antibody interaction. Indeed, almost 91% of antigen–antibody complexes in PDB were solved by X-ray crystallography^8^. However, X-ray crystallography is labor intensive, expensive and time consuming. Additionally, crystallization requires bringing the sample to highest possible concentration without causing aggregation, with success not being guaranteed. Moreover, information regarding binding energetics of individual epitope or paratope residues (hotspots) cannot be gleaned from X-ray structural data alone, requiring additional experimental analysis (e.g., site-directed mutagenesis)^9^. In recent years, molecular docking has emerged as a fast alternative route for studying the molecular basis of antigen–antibody interactions^10^. This growth was fueled by improvements to the docking methods (energetics based or machine learning based) and an increase in the number of solved protein complex structures and templates^11^. In particular, docking of antibody-antigen interactions has some exceptional advantages over traditional protein–protein docking owing to the highly conserved nature of the Ig fold which essentially restricts the binding surface to regions spanning/surrounding the complementarity-determining region (CDR) loops. Docking algorithms can be divided into two categories regarding the use of experimental data. The first category conducts an exhaustive search of different antigen–antibody configurations without using knowledge of interfacial residues, so-called ab initio docking algorithms. The second category consists of data-driven docking algorithms that make use of predicted or experimentally determined epitope and paratope constraints to guide the search process. For example, Tit-oon et al. employed a knowledge-based strategy to guide alanine scanning and computational docking, which led to the accurate prediction of an antigen-antibody interface^12^. Cannon et al. used experimental alanine scanning to optimize computational docking for *in-silico* affinity maturation of a high affinity antibody^13^. These studies have suggested that the success of molecular docking is critically impacted by use of experimental data.

Herein, we employ an integrated approach that combines alanine scanning and molecular docking to predict a model of FGF23-Burosumab interaction. The model explains the species cross-reactivity of Burosumab, viz., its weak binding towards mouse FGF23. Using the model, we reverse engineered mouse FGF23 with mutations from the human counterpart and successfully generated a variant that showed binding to Burosumab. Then, we computationally evaluated the effects of single mutations in the CDRs of Burosumab on binding to FGF23. This led us to engineer a double mutant having improved affinity to FGF23, thus validating the structural model.

## Results

### Paratope alanine scanning

Towards our aim of understanding how Burosumab interacts with FGF23, we first investigated the binding interface of Burosumab to select functional residues that may contribute to the binding. From amino sequence analysis, Burosumab possesses a hydrophilic CDR surface and a negatively charged HCDR3 with the net charge of -3 (Supplementary Fig. S2). In order to determine the critical paratope residues (hotspots) responsible for binding, we built a homology model of Burosumab Fv using Rosetta Antibody 3 module from ROSIE server (Supplementary Table S1). From the model, the fifty-six CDR residues that form the paratope structure were mutated to alanine using site-directed mutagenesis, expressed as Burosumab alanine mutants in HEK293T cells, and purified using Protein A chromatography.

**Figure 1:**
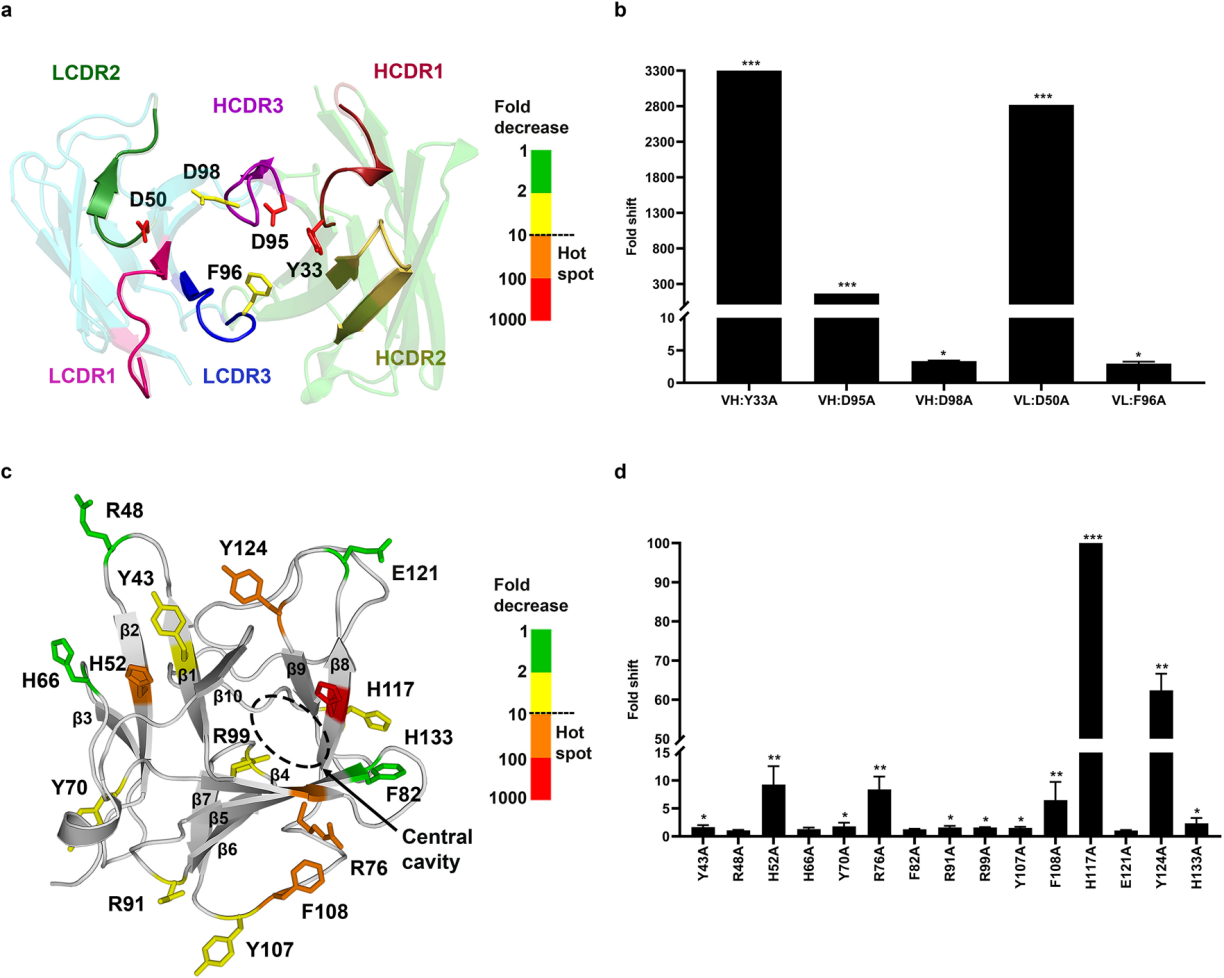
Paratope and epitope scanning for hotspot identification. Fifty-six Burosumab residues that were solvent exposed and covered all CDR loops were mutated to alanine and tested individually for binding to wild-type FGF23 using spot ELISA (Supplementary Tables S2 and S3). For each alanine mutant, the fold decrease in binding was calculated by comparing A450 of each mutant with wild-type Burosumab. The five key affinity altering residue positions (VH:Y33, VH:D95, VH:D98, VL:D50, VL:F96) were identified and mapped onto FGF23-binding interface (**a**). The residue coloring indicates the fold shift in binding relative to WT Burosumab: green: 1–2; yellow: 2–10; orange: 10–100; red: 100–1000. These five positions were considered as constraints for computational docking. (**b**) Fold decrease in binding for a representative set of mutations was shown. Degree of fold decrease was denoted by asterisk (*) as compared to the wild type: 2 to tenfold (*), > 10 to 100 fold (**), and > 100 fold (***). The homology model of Burosumab was built using Rosetta Antibody 3 provided by ROSIE server. CDR numbering was based on Chothia’s definition. For epitope scanning, fifteen solvent-exposed FGF23 residues that covered every domain of N-terminal domain of FGF23 were mutated to alanine and tested for binding to wild-type Burosumab. Fold decrease in binding was calculated from indirect ELISA of FGF23-Burosumab interaction by comparing a *K*_d_ value (nM) of each mutant with wild-type FGF23. Five epitope residues: H52, R76, F108, H117, Y124 with nearly a 10-fold decrease in binding or more upon alanine mutation were mapped onto FGF23 structures (**c**,**d**). These key epitope residues appeared to be in close proximity to the D2 and D3 domains of FGF23 receptor (FGFR) (Supplementary Fig. S1). N-terminal domain of FGF23 structure was sourced from PDB ID: 5W21. Structures were illustrated by PyMOL version 2.2.0. The graphs were created by GraphPad Prism version 8.4.3.

We next evaluated the effect of each Burosumab mutant on the FGF23-Burosumab interaction using ELISA (Supplementary Tables S2 and S3). On the heavy chain, we found two alanine mutations: Y33A (HCDR1) and D95A (HCDR3) that caused significant reduction in binding (> 100-fold decrease in *K*_d_), whereas another mutation D98A caused a mild reduction in binding (2.62-fold). Alanine mutations from HCDR2 did not significantly affect the binding. On the light chain, D50A (LCDR2) knocked out the binding (> 100-fold decrease in *K*_d_), while F96A (LCDR3) decreased the binding by 2.32-fold (Fig. 1a,b). Collectively, alanine scanning identified five paratope residues critical to the binding of which three residues: VH: Y33, VH: D95, and VL: D50 were considered as hotspots. We also observed that three out of five residues are negatively charged residues, suggesting that negatively charged residues in CDRs may play a dominant role in the binding and imply a likelihood for interacting with positively charged residues on the binding interface of FGF23. Of note, these critical residues were clustered at the VH – VL interface, suggesting that the concave surface formed by VH and VL domains may also play a key role in the recognition of FGF23 (Fig. 1a).

### Epitope alanine scanning

In order to determine the hotspots on the epitope, we first calculated the solvent accessibility of all FGF23 residues on the N-terminal domain to search for highly exposed FGF23 residues. The selection was predicated on the assumption that residues with high solvent accessibility were likely to participate to the binding interface with Burosumab. We used BIOVIA Discovery Studio version 2017 R2 on the crystal structure of FGF23 (PDB: 5W21) reported by Chen et al. (PDB: 5W21)^14^ to compute solvent accessibility. Residues with relative solvent accessibility (RSA) more than 20% of maximum RSA within the structure were designated as solvent exposed residues, consistent with published studies^12,15^. In total, we selected fifteen solvent exposed FGF23 residues that covered every sub-domain of FGF23. Specifically, the FGF23 residues Y43, R48, H52, H66, Y70, R76, F82, R91, R99, Y107, F108, H117, E121, Y124, and H133 were mutated to alanine using site-directed mutagenesis. The N-terminal domain of FGF23 mutants was attached to the His-SUMO tag (denoted as FGF23 in subsequent parts), expressed in BL21(DE3), purified using Ni–NTA resin, and calculated for their purity using SDS-PAGE analysis (Supplementary Fig. S3). All FGF23 mutants were coated on ELISA plate in proportion to their purity (Methods) and evaluated for binding to Burosumab (Fig. 1c).

Results revealed five hotspot residues that significantly impacted the binding upon modification. Notably, alanine substitution at H52, R76, F108 residues of FGF23 reduced binding ranging between 7 to 10-fold. Y124A mutation reduced binding by almost 50-fold as compared with the wild-type FGF23 (Fig. 1d). Of note, H117A mutation alone completely knocked the binding (> 100-fold). We also noticed that 3/5 of FGF23 hotspot residues were positively charged as opposed to the negatively charged hotspot residues on the paratope interface, suggesting that FGF23-Burosumab interaction may be driven by electrostatically charge-charge complementary. Unexpectedly, we found that these epitope hotspots were located on two different regions, although they lined close to the central cavity of FGF23 overlapping with the FGFR binding site (Fig. 1c). The first region included H52 on β2 strand and Y124 on β9 strand, which seemed to be in close contact with the D2 domain of FGFR and the second region included R76 on β4 strand, F108 on β7-β8 hairpin loop, and H117 on β8 strand, which are in proximity to the D3 domain of FGFR (Supplementary Fig. S1). Taken together, epitope alanine scanning identified five potential epitope hotspots: H52, R76, F108, H117, and Y124 overlapping with FGFR binding interface of FGF23.

### Molecular docking of FGF23 and Burosumab

**Figure 2:**
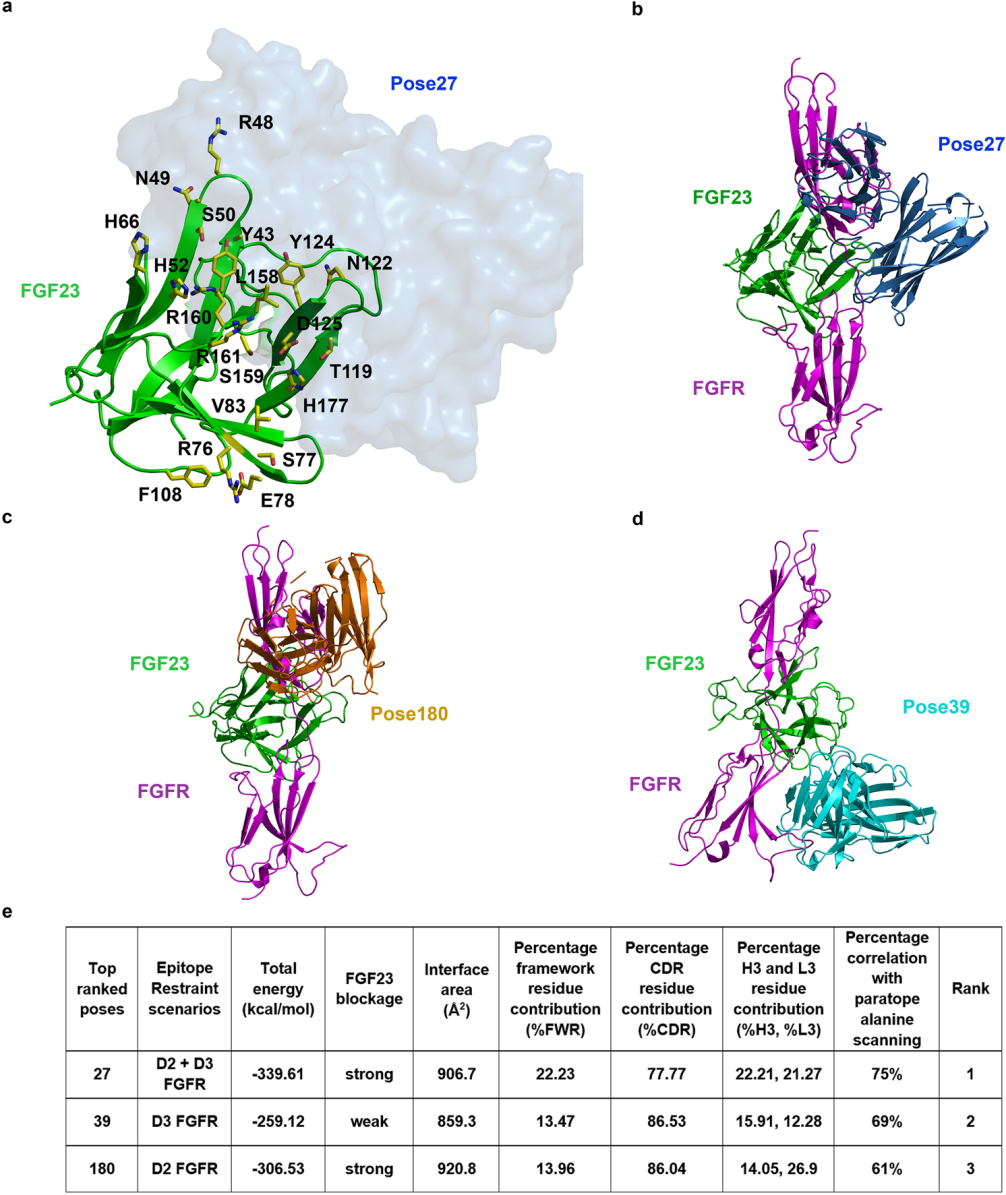
Computational docking and model assessment. HADDOCK2.4 was run using default parameters with the homology model of Burosumab Fv (RosettaAntibody3) and the unbound crystal structure of FGF23 (PDB ID: 5W21). The identified paratope and epitope residues from alanine scanning were used as active residue restraints during the docking process. Pose27 was seen to interact with a number of FGFR-interacting FGF23 residues (yellow sticks), highlighting the FGF23:FGFR blocking potential of the antibody (**a**). The top pose from each docking scenario was superimposed with FGF23-FGFR complex (PDB ID: 5W21) using PyMOL version 2.20 (**b**–**d**). Table in panel (**e**) shows the assessment of the top three poses using the seven structural criteria. Amongst the three poses, pose27 had the most favorable structural characteristics: (1) high FGF23 blockage, (2) the lowest binding energy, (3) the best correlation with paratope alanine scanning data, (4) acceptable interface area (> 800 Å^2^), (5) reasonable %FWR for lock-and-key binding given a comparable size between FGF23 and Burosumab Fv, (6) acceptable percent contribution of CDR residues at the interface (%CDR), and (7) highest contribution of H3 and L3.

To determine how Burosumab interacts with FGF23, we docked the structure of FGF23 (source: PDB: 5W21) with the homology-based Fv model using the HADDOCK2.4 docking server (https://wenmr.science.uu.nl/haddock2.4/). The identified paratope and epitope hotspot residues from alanine scanning were used as restraints during the docking process. HADDOCK2.4 has been validated on benchmark version 5.0 (BM5) datasets, which is comparable with other docking programs evaluated by CAPRI studies^16,17^. Since the epitope hotspots mapped to distinct regions of FGF23 placing them in close proximity to D2 or D3 subunits of FGFR, we performed docking with three different sets (scenarios) of epitope residue restraints: (1) H52 and Y124 residues (residues contacting D2-FGFR), (2) R76, H117, and F108 residues (residues contacting D3-FGFR), and (3) H52, Y124, R76, H117, and F108 residues (combined D2 and D3 scenarios), while the paratope residue restraints: VH: Y33, VH: D95, VH:D98, VL:D50, and VL:F96 were held constant. HADDOCK2.4 takes input from experiments including site-directed mutagenesis to guide the docking process. The interface is selected based on a combination of traditional energetics and shape complementary metrics. Notably, the HADDOCK score is calculated based on interaction energy that includes van der Waal energy, electrostatic energy, empirical desolvation energy and ambiguous interaction restraints (AIR)^18^. We ran HADDOCK2.4 under a default setting, which generates 1,000 poses in total for each run. In addition to employing the interfacial mutations to guide the docking process, we used knowledge-based physicochemical constraints to filter and rank order the predicted models.  Briefly, the top pose from each scenario was analyzed using seven criteria: (1) degree of stereo blockage of FGFR binding, (2) binding energy, (3) percent correlation with paratope alanine scanning data, (4) buried surface area at the interface, (5) percent contribution of framework residues (%FWR) at the interface, (6) percent contribution of CDR residues at the interface (%CDR), and (7) percent contribution of HCDR3 domain (%H3) at the interface (Supplementary Fig. S4). Notably, structural analysis of antigen–antibody structural complexes from PDB have established upper bound on features 4–7, which helped eliminate models that significantly deviate from native-like structures. Interestingly, we found that the top-four poses within each scenario shared similar binding interface. The top poses from the three scenarios were compared using the seven physicochemical criteria to determine which pose (or epitope-restraint scenarios) gave the most favorable native-like interface (Fig. 2).

Pose27 and pose180 exhibit a strong blockage of FGFR with similar buried surface areas (906.7 and 920.8 Å^2^, respectively) whereas pose39 possesses a slightly smaller buried surface area (859.3 Å^2^). To investigate residue contributions at the interface, each pose was submitted to PISA server (https://www.ebi.ac.uk/pdbe/pisa/). Interfacial data of pose27 showed that 77.77% of interface area was contributed by CDR residues of which HCDR3 (H3) and LCDR3 (L3) residues accounted for 22.21% and 21.27%, respectively while the remaining four CDRs account for 34.29%. This suggests that pose27 had a relatively high contribution of HCDR3 and LCDR3 to the binding, as commonly observed in other antigen–antibody interfaces^19–21^. Unlike pose27, pose39 and pose180 showed relatively smaller HCDR3 contribution (pose27: 22.21%; pose39: 15.91%; pose180: 14.05%), even though they both had higher overall CDR contribution than pose27 (pose27: 77.77%; pose39: 86.53%; pose180: 86.04%). Interfacial data also showed that pose27 had the highest % FWR contribution (22.23%), which was accounted by residues flanking HCDR1 (Y33), HCDR2 (I50, T58, and S59), and LCDR2 (Y49). Most importantly, pose27 showed the highest % correlation with paratope alanine scanning, which was followed by pose39 and pose180.

Taken together, we considered pose27 as the top-ranked pose as it best agreed with (1) experimental data (highest % correlation with paratope alanine scanning), (2) physiochemical data, (3) biological data (strong blockage of FGF23), and (4) immunological data (high HCDR3 contribution which is a characteristic of therapeutic antibodies having high specificity and affinity for the target epitope; Burosumab is a high affinity antibody with a reported *K*_d_ of 10^–11^ M).

**Figure 3:**
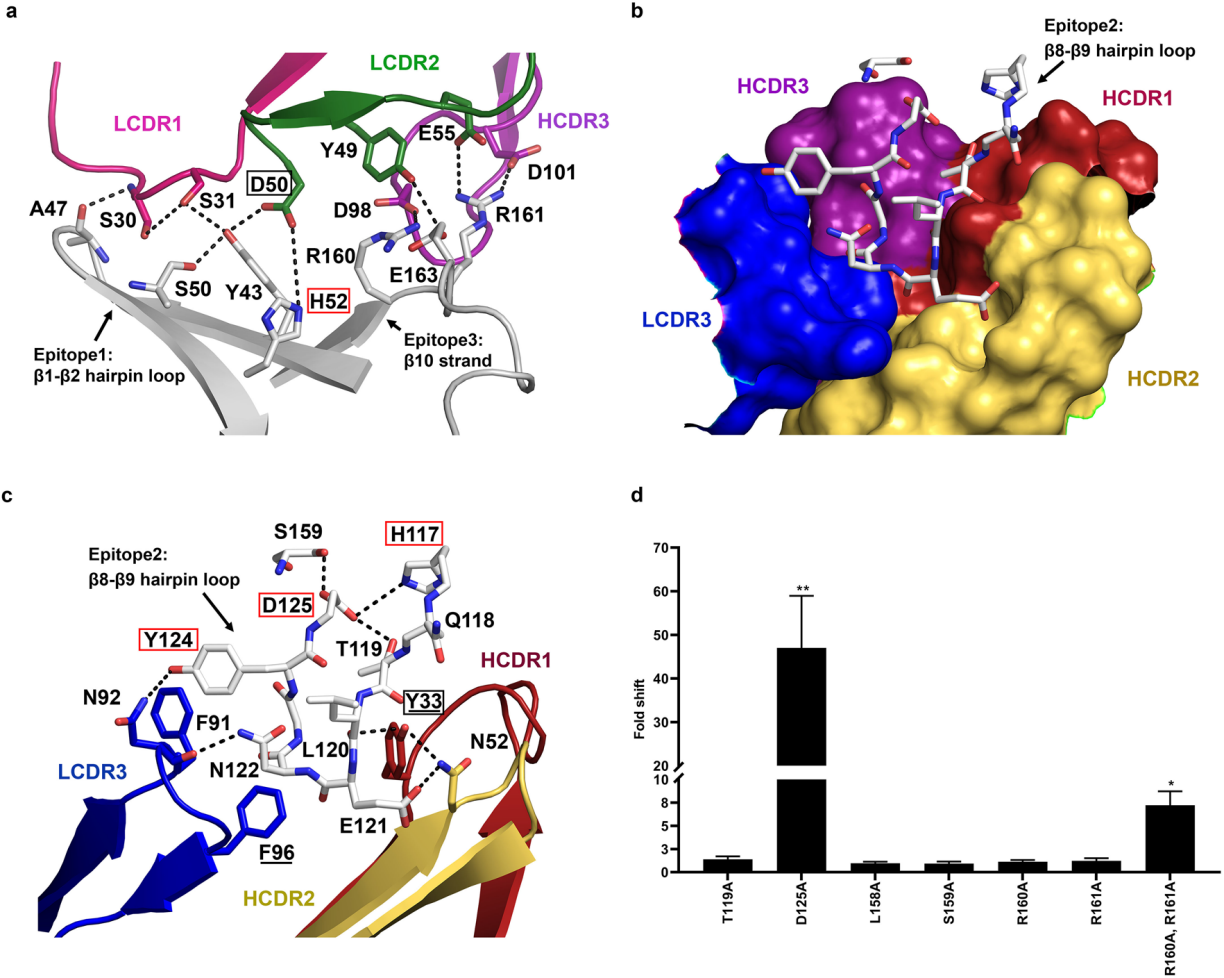
Close-up views of key epitope-paratope interacting residues over the three main epitope regions on FGF23 based on pose27 and its validation. (**a**) The backbone and side chains of FGF23 are colored in grey. Cartoons representing LCDR1 (pink) and LCDR2 (green) that contributed to the interaction by forming multiple hydrogen bonding networks (dash line) with β1-β2 hairpin loop (white) of FGF23 (epitope-1). HCDR3 (purple) interacted with β10 strand of FGF23 (epitope-3). Epitope and paratope hotspots are shown in red and black boxes, respectively. (**b**) Cartoon representation of the β8-β9 hairpin loop of FGF23 (epitope-2) forming a sharp turn for protruding into the pocket of Burosumab (surface representation), which showed its direct contacts with LCDR3 (blue), HCDR1 (red), and HCDR2 (yellow)—HCDR3 (purple) allosterically affects the formation of this pocket. (**c**) Two paratope residues: VL:F96 and VH:Y33 (hotspots) (as underlined) are necessary for the pocket formation for interacting with the β8-β9 hairpin loop. While three epitope hotspots residues: H117, Y124, and D125 of FGF23 (red box) determine a restricted/sharp turn conformation of β8-β9 hairpin loop. (**d**) Seven additional FGF23 alanine mutants: T119A, D125A, L158A, S159A, R160A, R161A, and combined R160A and R161A were created to prove the validity of pose27. ELISA was used to measure a *K*_d_ value of these FGF23 alanine mutants in comparison with the wild-type FGF23. Degree of fold decrease was denoted by asterisk (*) as compared to the wild type: 2 to 10-fold (*) and > 10 to 100-fold (**). D125A and combined R160A and R161A mutations decreased the binding between > 10 to 100-fold and 2 to 10-fold, respectively. Bar plot was created by GraphPad Prism version 8.4.3.

Pose27 showed that Burosumab recognized three main regions on FGF23: β1-β2 hairpin loop (epitope-1), β8-β9 hairpin loop (epitope-2), and β10 strand (epitope-3) by forming multiple hydrogen bond interactions, salt bridges, pi-anion interactions, and attractive charge interactions (Supplementary Table S4). Interfacial analysis showed that epitope-1 and epitope-3 of FGF23 together formed a planar binding site for Burosumab in which A47 and Y43 of the epitope-1 formed hydrogen bonding networks with LCDR1 and LCDR2, while R160 and R161 of the epitope-3 formed both salt bridge and attractive charge interactions with LCDR2 and HCDR3 (Fig. 3a). On the contrary, epitope-2 formed a conformational binding site (sharp turn) for protruding into a pocket region at the VH-VL interface of Burosumab, which was formed mainly by LCDR3, HCDR1, and HCDR2 (Fig. 3b). We speculated that H117, T119, D125, and S159 were four key FGF23 residues that maintain the structural shape of epitope-2 through intra-interaction networks (Fig. 3c), while R160A and R161A were two key residues that stabilized the complex of the interaction.

To validate pose27, six additional FGF23 alanine mutants: T119A, D125A, L158A, S159A, R160A, and R161A deemed important from structural analysis were generated and tested for binding to Burosumab (Fig. 3d). Specifically, T119A, D125A, and S159A mutations were selected to see the effect of the mutation to the intra-interaction networks, which determines the sharp turn conformation of β8-β9 hairpin loop or epitope-2 (Fig. 3c). On the other hand, L158A, R160A, and R161A mutations on β10 strand or epitope-3 were selected to investigate the effect of the mutation on the hydrophobic network at the center of interface (by L158A) and the electrostatic interaction observed at the peripheral interface (by R160A and R161A) (Supplementary Table S4).

Among these mutants, D125A mutation remarkably reduced the binding consistent with the structural model that showed the intra-electrostatic interaction between D125 and H117 (hotspots) critically governs the sharp turn of β8-β9 hairpin loop. Intriguingly, the other single mutations caused no significant change to the binding. Notably, R160 and R161 which appeared to be involved in electrostatic interactions did not drop binding upon mutation to alanine. We speculated that the proximity of these residues may render one to compensate for the other. To test this hypothesis, we created a double mutant—R160A-R161A—and tested its binding to Burosumab. The double alanine mutant dropped the binding by 7-fold, suggesting that each of these residues in fact play a compensatory role in the absence of the other (Fig. 3d).

### Assessment of antibody cross-reactivity

**Figure 4:**
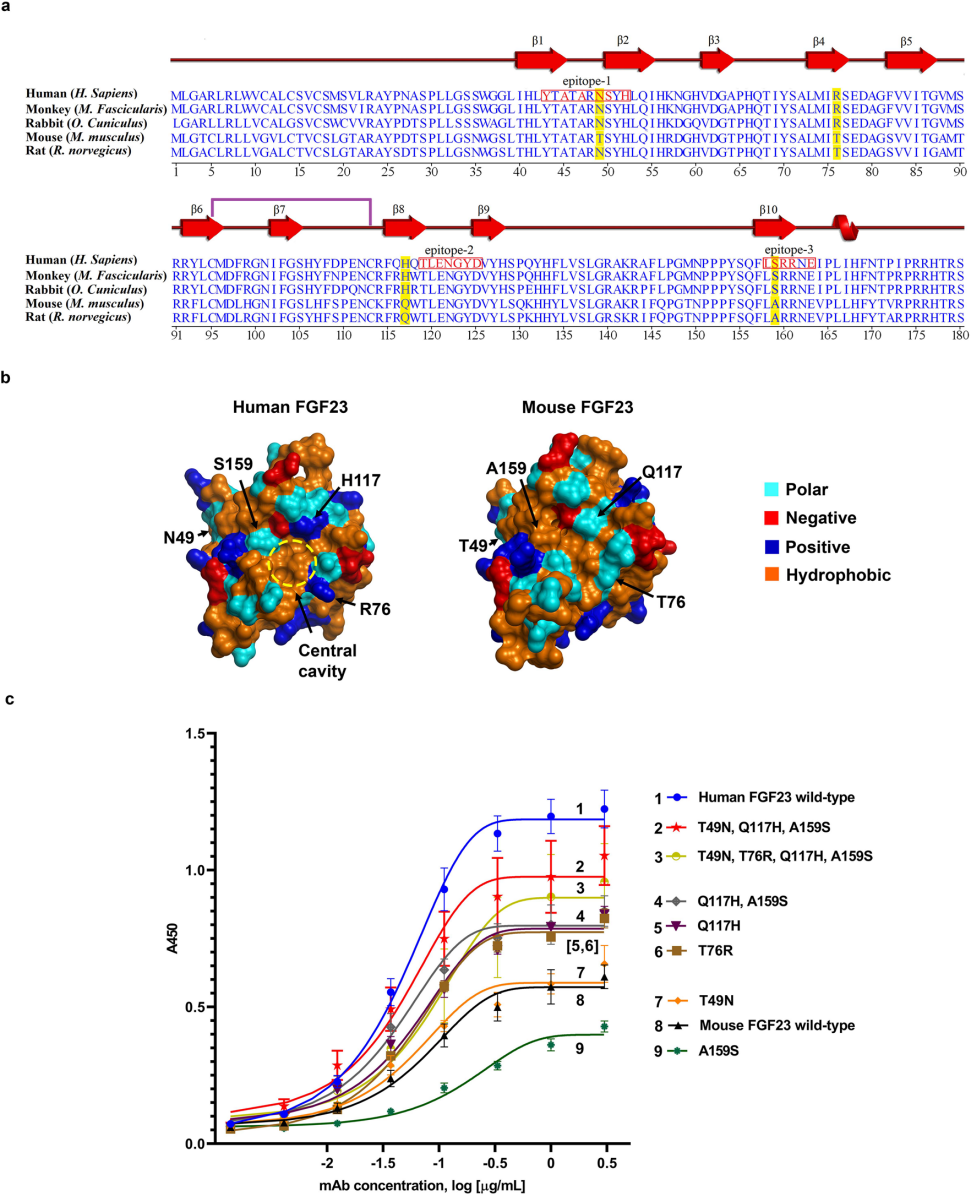
Conservation of antibody epitope in different species and reverse engineering of murine FGF23 to bind Burosumab. (**a**) The amino acid sequence alignment of FGF23 across binder (monkey and rabbit) and non-binder species (mouse and rat) is shown in a clustal omega format. The three distinct epitope regions (red box) were well conserved across, albeit with key species-specific differences. The residues that differentiate those binder species (monkey and rabbit) from non-binder species (mouse and rat) are shaded in yellow. The residues that make contact with Burosumab based on pose27 are colored in red. Secondary structures: β strand, α-helix, and a single disulfide formation between cysteine 93 and 113 are annotated as red arrow, red helix, and purple line, respectively. (**b**) The four putative cross-species determining residues: N49, R76, H117, and S159 (human FGF23 numbering) were mapped onto the surface of human and mouse FGF23. (**c**) Binding activity of mouse FGF23 mutants with reverse mutations measured by indirect ELISA. Combination of three reverse mutations: T49N, Q117H, and A159S was able to rescue mouse FGF23 binding to Burosumab nearly similar to the human FGF23 wild type. Curves were drawn by GraphPad Prism version 8.4.3. A450 refers to absorbance at 450 nm. Structures were created by PyMOL version 2.2.0.

Next, we sought to employ our model to explain the cross-species reactivity of Burosumab. As reported by the originator in the European Medicines Agency (EMA), Burosumab binds to FGF23 from human, monkey, and rabbit species (so-called binder species), but not from mouse and rat species (so-called non-binder species)^2^. We first performed amino acid sequence alignment of N-terminal domain of FGF23 from human, monkey, rabbit, rat, and mouse. The FGF23 from the different species exhibited varying degrees of sequence identity to human FGF23: rabbit: 87%; monkey: 88%; mouse: 78%; and rat: 78%. We identified thirty-three residues that were different between human and mouse FGF23 within the N-terminal domain. These residues include three residues: I40, N49, K57 on β1-β2 hairpin loop, two residues: N58 and A64 on β3 strand, four residues: R76, F82, V88, S90 on β4-β5 hairpin loop, five residues: Y93, R99, H106, Y107, D109 on β6-β7 hairpin loop, nine residues: R114, Q116, H117, Q118, H128, P130, Q131, Y132, F134 on β8-β9 hairpin loop, five residues: A144, L146, M149, Y154, S159 on β10 strand, and five residues: I164, I167, N170, P172, I173 on α-helix region (Fig. 4a). Seventeen out of thirty three (17/33) or 52% of these residues are located on β1-β2 hairpin loop, β8-β9 hairpin loop, and β10 strand, which make up the predicted epitope regions.

These findings prompted us to investigate the underlining mechanism of the cross-reactivity of Burosumab using pose27. We hypothesized that the physiochemical properties of the central cavity, which plays a central role for binding recognition of Burosumab, may also contribute to the species-specific binding behavior (Fig. 4b). To test this hypothesis, we created four back mutations in which polar and hydrophobic residues surrounding the central cavity of mouse FGF23: T49, T76, Q117, and A159 were replaced by the corresponding charged and polar residues of human: N49, R76, H117, and S159, respectively. Three combined mutations: (1) combined Q117H and A159S mutations, (2) combined T49N, Q117H, A159S mutations, and (3) combined T49N, T76R, Q117H, A159S mutations were also created. These seven mouse FGF23 mutants were expressed and tested their binding to Burosumab using ELISA (Fig. 4c). Among those individual mutations, both Q117H and T76R mutations slightly improved the binding as compared to wild-type human FGF23 and mouse FGF23, while T49N mutation exhibits similar binding to wild-type mouse FGF23 and A159S mutation worsens the binding. Amongst the three combination mutations, Q117H and A159S mutations and T49N, Q117H, A159S mutations significantly rescued the binding nearly equivalent to wild-type human FGF23. These results suggest that the co-occurrence of H117 and S159 residues was necessary for the binding to mouse FGF23, whereas R76 does not play a significant role to the binding. Intriguingly, the originator did not detect binding between Burosumab and murine FGF23 by competitive SPR. On the other hand, we found that Burosumab bound weakly to murine FGF23 on an ELISA. This disparity may arise from different formats of the binding assay. In particular, competitive SPR format has a higher stringency as compared with the ELISA format which permits avidity effects from the bivalent binding of Burosumab.

### Affinity enhancement of Burosumab

**Figure 5:**
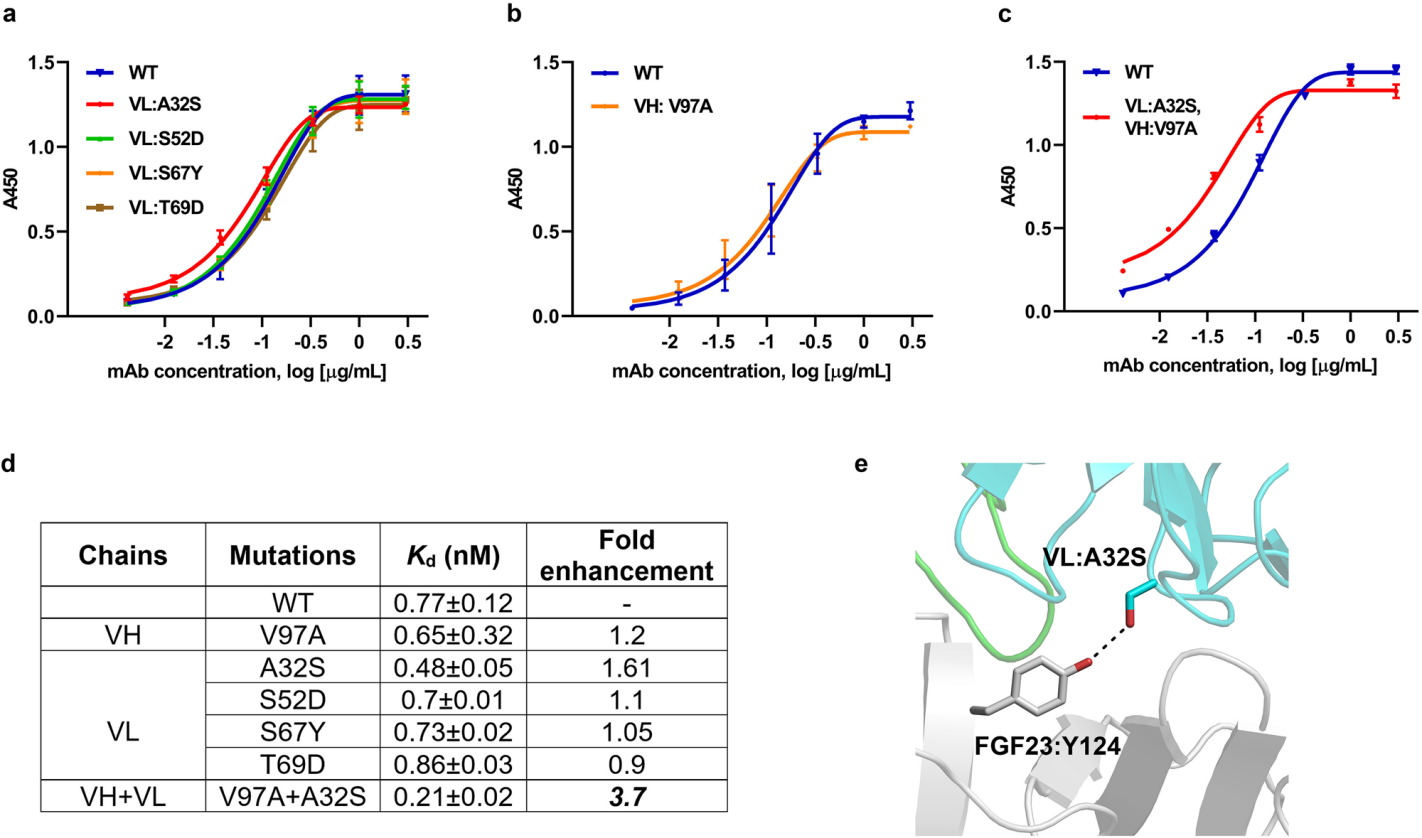
Binding activity of Burosumab variants designed to enhance affinity based on pose27. Four mutations on the light chain were created and tested for binding to wild-type FGF23 using ELISA (**a**). Only A32S mutations showed 1.6-fold affinity enhancement based on *K*_d_ value compared with wild-type Burosumab. V97A, which showed small affinity enhancement (1.2-fold) from alanine scanning promoted the binding affinity up to 3.7-fold when combined with A32S (**b**,**c**,**d**). VL:A32S is predicted to form a new favorable hydrogen bond formation with Y124 of FGF23 (**e**). Curves were drawn by GraphPad Prism version 8.4.3. A450 refers to absorbance at 450 nm. Structure was created by PyMOL version 2.2.0.

The structural insights of FGF23-Burosumab interaction gained from computational docking prompted us to further design Burosumab variants with improved binding affinity. Using pose27 as the starting model, four mutations on VL domain – A32S, S52D, S67Y, T69D – were predicted to enhance the interatomic interactions with the target epitope. Notably, VL:S52D and VL:T69D mutations were aimed to enhance electrostatic interactions with R160 and R48 of FGF23, respectively. On the other hand, VL:A32S and VL:S67Y mutations were designed to enhance hydrogen bond formation with Y124 and N49 of FGF23, respectively. The four single mutants were generated and tested for their binding to FGF23 as compared with wild-type Burosumab using ELISA. Binding results showed that only VL: A32S had noticeably enhanced affinity by 1.6-fold (Fig. 5a). To further enhance the affinity of Burosumab, we combined VL: A32S mutation with VH: V97A mutation. The latter mutation improved binding affinity (1.2-fold) in paratope alanine scanning (Fig. 5b). This combined Burosumab variant (combined VH: V97A and VL: A32S mutations) showed a substantial 3.7-fold improved binding as compared with the wild-type antibody (Fig. 5c,d).

 We believed that 3.7-fold enhancement may arise from entropy lost-enthalpy gain compensation; in that the entropy lost from the decrease of flexibility of V97A upon complex formation was compensated by the enthalpy gain of A32S due to more complex stabilization by inter-hydrogen bond formation between A32S and Y124 of FGF23 (Fig. 5e). These findings suggest that CDR modifications with least overall structural/energetic disturbance could form an underlying strategy for affinity enhancement of therapeutic antibodies that possess rigid/densely packed CDRs.

## Discussion

Understanding the molecular basis for antibody-antigen interaction is important for successful design of therapeutic antibodies. In this study, we employed molecular docking driven by alanine scanning to predict a model of interaction between Burosumab and human FGF23. Briefly, alanine scanning was employed to determine critical paratope and epitope hotspots, which then guided the molecular docking process to predict the 3D model. Our top-rank model demonstrated that the light chain of Burosumab mediated the binding over a planar surface of FGF23 covering β1, β2, and β10 strands, while the heavy chain recognized a sharp turn of β8-β9 hairpin loop, a region near the central cavity of FGF23. Through this model, we reconciled the cross-species reactivity of Burosumab against the different species and further designed a 3.7-fold affinity-enhanced variant of Burosumab.

We found that the interaction between FGF23 and Burosumab was dominated by electrostatic charge-charge interactions; indeed this was supported by the Ala-scan hotspot analyses, which identified 3 (out of 5) negatively charged paratope and 3 (out of 5) positively charged epitope residues. It has been known that high affinity antibodies often possess a rigid CDR conformation through several rounds of natural antibody maturation^22^. The number of intra-electrostatic interactions and preference of polar and charge side chains at the CDR loops were reported to be the key indicative factors of rigid CDR conformation and antibody specificity^23,24^. The presence of two intra-electrostatic interactions within the HCDR3 of Burosumab, including VH:R94-VH:D101 and VH:H35-VH:D95 and the enrichment of polar and charged residues in CDRs (53% vs 63% of HyHEL26- mAb with known rigid CDRs^23^) suggest rigid conformation of Burosumab in accordance with the previous studies. Bedouelle et al. found that paratope hotspots tend to cluster at the diversity and junction regions (or HCDR3 and LCDR3 loops) as evident in broadly neutralizing antibodies such as mAb4E11^25^. The authors correlate the clustering of paratope residues to CDR rigidity. In a similar manner, we observed a number of critical paratope residues lined within the diversity and junction regions: VH:D95 (hotspots) and VH:D98 in HCDR3 and VL:F96 in LCDR3. With these results, we believed that Burosumab binds to FGF23 in the lock-and-key manner through a pre-organized CDR conformation. This conformational specificity of Burosumab was consistent with knockout (hotspots) mutations on D125 or H117 (Figs. 1d and 3d), which form electrostatic interactions with each other to maintain a sharp turn of β8-β9 hairpin loop, which is critical for Burosumab binding.

Several studies have reported the preference of aromatic residues at the paratope interface, which play a shape-complementary role by interacting with the backbone atom of the antigen^26,27^. In accordance with previous reports, we found that VL:F96 on LCDR3 and VH:Y33 (hotspots) on HCDR1 of Burosumab interact with backbone atom of β8-β9 hairpin loop of FGF23 in the shape-complementary manner with a specific CDR conformation (Fig. 3b,c). Amongst the two aromatic residues of FGF23: Y43 and Y124 identified in this study, only Y124A mutation had dramatic effect on the binding, while Y43A did not. The fact that Y124 (epitope hotspot) is located in the β8-β9 hairpin loop strengthens the importance of this area as the main epitope region for Burosumab. These findings indicate the role of aromatic residues in determining the shape-complementarity for FGF23-Burosumab interaction, which support the conformation-specificity of Burosumab.

In this study, the recognition of the specific conformation of FGF23 by Burosumab correlated with the cross-species reactivity of Burosumab. Upon amino acid sequence alignment of FGF23 in different species, majority of amino acid residues that differentiate between the binder FGF23 species and the non-binder FGF23 species lined within β8-β9 hairpin loop or in between the regions flanking β8-β9 hairpin loop. We showed that back mutations: Q117H and A159S of mouse FGF23 residues near the central cavity of FGF23 to corresponding human residues regained the binding activity of mouse FGF23 nearly equivalent to human FGF23 (Fig. 4c). The results not only support β8-β9 hairpin loop as the main structural epitope for Burosumab, but they also highlight the cooperative effects the residues have on each other.

In summary, we present an integrated approach combining molecular modeling, site-directed mutagenesis, human-guided design incorporating feedback from experiments for studying the structural basis of FGF23-Burosumab interaction. Our study provides key insights of the interaction and highlighted the underlying determinants of affinity and specificity of Burosumab, which can be used for rational engineering.

## Methods

### Recombinant His-SUMO-FGF23 expression and purification

DNA fragment of N-terminal domain of FGF23 with N-terminal His-SUMO tag according to FGF23 amino acid sequence on Uniprot (Q9GZV9) was synthesized by Integrated DNA Technologies and cloned into pET-28a (+) (Novagen) at *Xho*I and *Nco*I sites. Positive clones were screened by restriction enzyme digestion and further confirmed by sequencing. Expression of His-SUMO-FGF23 was carried out in BL21 (DE3) (Thermo Scientific) grown at 37 °C in Luria–Bertani (LB) broth (Sigma Aldrich) supplemented with 50 μg/mL kanamycin. The culture was induced at OD_600_ of 0.4 by 0.1 mM IPTG at 22 °C for 8 h. Culture was harvested and lysed with French press operated at 900 psi. Purification of His-SUMO-FGF23 was carried out using Amintra Ni–NTA affinity resin (Expedeon). The resin was washed with 10 mM imidazole in lysis buffer and the bound protein was eluted with 200 mM imidazole in lysis buffer, and buffer exchanged to phosphate buffered saline (PBS) using Amicon Ultra Centrifugation Filters 3 K (Merck). Protein concentration was measured using Nanodrop. Purity of proteins was determined by SDS-PAGE.

### Burosumab expression and purification

pcDNA 3.3(-) expression vectors encoding Burosumabs was transiently transfected using Polyethyleneimine Max (PEI-MAX, PolySciences) into HEK293-F FreeStyle cells grown in FreeStyle 293 Expression Medium (Invitrogen). The culture was maintained at 37 °C, 80% humidity and 8% CO_2_ for six days. Burosumabs were then purified using Pierce Protein A agarose (Thermo Fisher Scientific). The bound Burosumab was eluted with 0.1 M glycine pH 3.5 and neutralized with 1 M Tris pH 8.0. Fractions containing Burosumab were then pooled and buffer exchanged into PBS using Amicon Ultra Centrifugation Filters 30 K (Merck). Burosumab concentration was measured using NanoDrop™ 2000 (Thermo Fisher Scientific). Purity of antibody was determined using SDS-PAGE.

### Site-directed mutagenesis

Site-directed mutagenesis (SDM) was conducted to get twenty-two alanine mutations of human FGF23, seven mutations of mouse FGF23, thirty of antibody heavy-chain, and twenty-six of antibody light-chain mutants. PCR amplification reactions were conducted using a QuikChange Lighting Site-Directed Mutagenesis Kit (Agilent) according to the manufacture’s suggestion. PCR products were then digested with *Dpn*I for 30 min at 37 °C before transformed into *Escherichia coli* DH5α with 50 mg/mL ampicillin selection. Plasmid DNA was extracted from an overnight culture using plasmid DNA preparation kits (Invitrogen). Positive colonies were confirmed for the presence of the desired mutations by DNA sequencing.

### Enzyme-linked immunosorbent assay (ELISA)

Wild-type FGF23 or its alanine mutants were coated onto 96-well Maxisorp plates (Nunc) at a concentration of 2 μg/mL and incubated at 4 °C overnight. Plates were washed with PBS with 0.05% tween (PBST) prior to incubation with 200 μL of 1% BSA in PBST for 1 h. at room temperature, and followed by washing with PBST. Wild-type Burosumab or its alanine mutants were three-fold serially diluted in PBS starting at 9 µg/mL prior to adding into plates and incubating at room temperature for 2 h. Plates were washed again with PBST, and then 100 μL of goat anti-human IgG H&L conjugated with HRP (1:10,000, Abcam) was added to plates and incubated at room temperature for 1 h. After washing with PBST, the plate was incubated with 100 μL of TMB substrate (KPL) at room temperature for 3 min, and quenched with 100 μL of 1 N H_2_SO_4_. The absorbance was measured at 450 nm using a Spectramax S5 plate reader (Molecular Devices). Note that spot-check ELISA was performed in the same manner except that only two concentrations (0.04 and 1.2 µg/mL) of Burosumab were applied.

### Homology modeling of Burosumab variable region (Fv)

The structure of Burosumab Fv was built using Rosetta Antibody 3 from ROSIE server^28^. The Rosetta Antibody 3 generated an antibody structure by modeling each CDR (L1, L2, L3, H1, H2, and H3) and each framework (FRL and FRH) separately based on the most sequence-homologous templates (Supplementary Table S1). Each modeled CDR and FR was subsequently assembled with different VH/VL orientations, resulting in ten distinct crude structures. Finally, H3 was re-modeled again using a kinematic loop modeling algorithm together with VL/VH orientation to fit the modeled H3 loop. During this step, the side chains and loop backbones for each CDR were also refined simultaneously^29,30^. Burosumab possessed the L1, L2, L3, H1, and H2 CDR loops belonging to canonical classes 2, 1, 3, 1, and 3, respectively. H3 does not belong to any known canonical class (Supplementary Fig. S2 and Table S1).

### Molecular docking using HADDOCK2.4

The homology-modeled Burosumab heavy and light chains were combined into one single chain numbering using pdb-tools scripts and used as a receptor for docking^31^, while the solved crystal structure of N-terminal domain of FGF23 retrieved from the co-crystal structure of FGF23:α-Klotho:FGFR complex (PDB: 5W21) was used a ligand for docking. The structure was added for the missing residues and cleansed by removing water molecules and bound ligands using PyMOL 2.2.0 prior to uploading into HADDOCK2.4 docking server^18^. HADDOCK2.4 is a semi-flexible docking that exploits experimental data such as mutagenesis data to guide docking process along with energy functions and shape complementarity to predict a protein–protein interaction. HADDOCK2.4 docks proteins by first sampling the conformational space based on the input residue restraints using rigid shape complementarity and rigid body energy minimization to optimize the interaction. Next, flexibility of both backbone and side chain of residues at the interface were introduced, which allow conformational changes to optimize the interface packing. Then, the docking solutions were refined with explicit solvents to improve energetics of the interaction. Finally, HADDOCK2.4 returned the top clusters ranked by the average HADDOCK scores of the top four poses in each cluster. Each top pose from each top cluster was then assessed for agreement with stereo-blockage of FGF receptor and paratope alanine scanning data. The top-ranking pose was then selected for further analysis of interacting residues using PISA server^32^ and BIOVIA Discovery Studio version 2017 R2.

## Supplementary Information

Supplementary Information.

## Data Availability

All data generated or analysed during this study are included in this published article (and its Supplementary Information files).
