# Supplementary material for: Prediction of the structural interface between fibroblast growth factor23 and Burosumab using alanine scanning and molecular docking
Source: Sci Rep. 2022 Aug 30;12:14754. doi: 10.1038/s41598-022-18580-3 (PMC9427789; doi:10.1038/s41598-022-18580-3)
Supplement: Supplementary file 1 — Supplementary Information. [file 41598_2022_18580_MOESM1_ESM.pdf]

Supplementary Information

Title

Prediction of the structural interface between fibroblast growth factor23 and Burosumab using alanine scanning and molecular docking

Authors

Karnpob Kanhasut[1], Kannan Tharakaraman[2], Mathuros Ruchirawat[3,4], Jutamaad Satayavivad[4,5,6], Mayuree Fuangthong[1,3,4,*], Ram Sasisekharan[2,7,*]

Affiliations

[1]Program in Applied Biological Sciences, Chulabhorn Graduate Institute, 54 Kamphaeng Phet 6 Rd, Bangkok, 10210, Thailand.

[2]Koch Institute for Integrative Cancer Research, Massachusetts Institute of Technology, Cambridge, MA 02139, USA.

[3]Translational Research Unit, Chulabhorn Research Institute, Bangkok 10210, Thailand.

[4]Center of Excellence on Environmental Health and Toxicology (EHT), OPS, MHESI, Thailand.

[5]Laboratory of Pharmacology, Chulabhorn Research Institute, 54 Kamphaeng Phet 6 Rd, Bangkok, 10210, Thailand.

[6]Program in Environmental Toxicology, Chulabhorn Graduate Institute, 54 Kamphaeng Phet 6 Rd, Bangkok, 10210, Thailand.

[7]Department of Biological Engineering, Massachusetts Institute of Technology, Cambridge, MA 02139, USA.

[*]rams@mit.edu, mayuree@cri.or.th

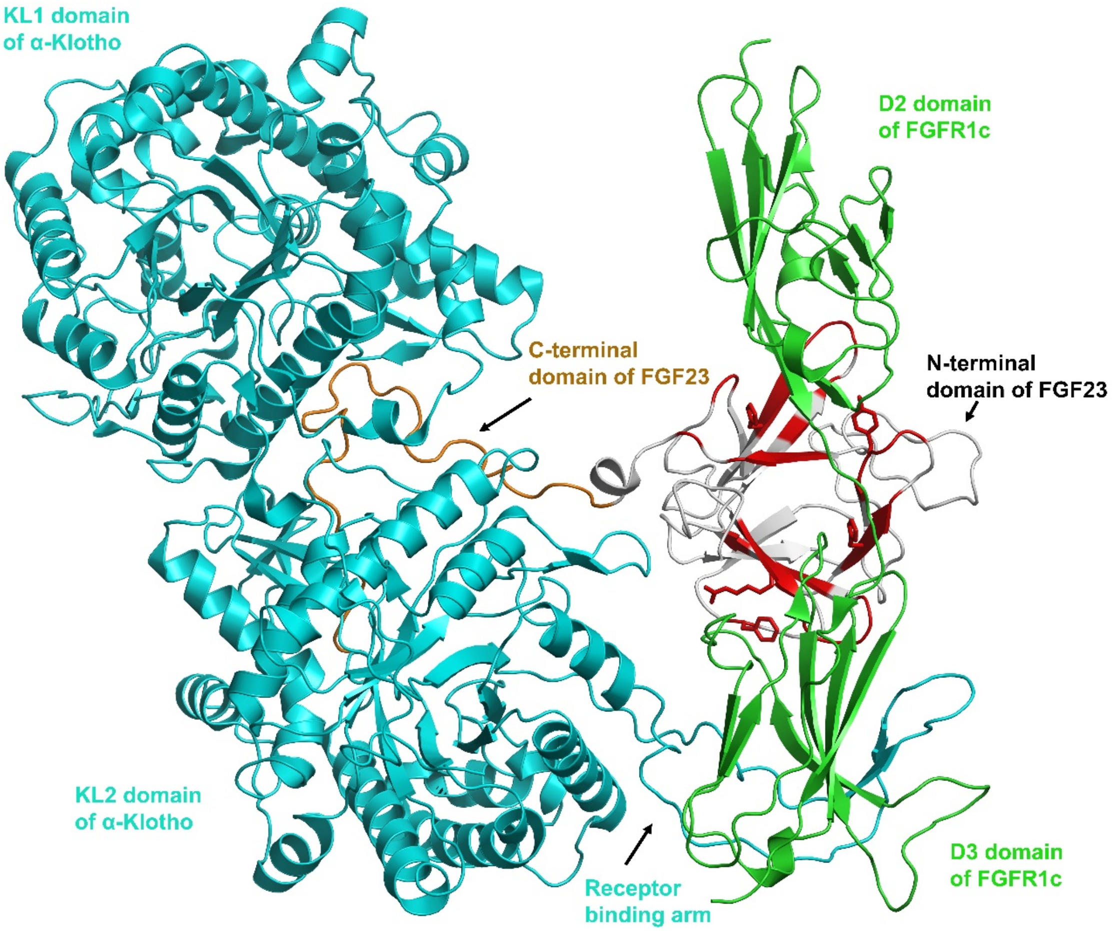

Supplementary Figure S1. Tertiary complex of FGF23, FGFR1c, and α-klotho. α-klotho (cyan) acts as an engager by simultaneously binding the D3 domain of FGFR1c (green) using the receptor-binding arm (RBA) and the C-terminal domain of FGF23 (orange) using the interface between KL1 and KL2 domain. α-klotho stabilizes FGFR1c-FGF23 interaction to promote metabolic signaling for phosphate re-uptake in the kidney tubular cells. The FGFR-binding regions of FGF23 (white) are highlighted in red color. The five epitope hotspots for Burosumab identified from alanine scanning (Fig. 1d) are shown as red sticks. The complex was sourced from PDB ID: 5W21 and created by PyMOL version 2.2.0.

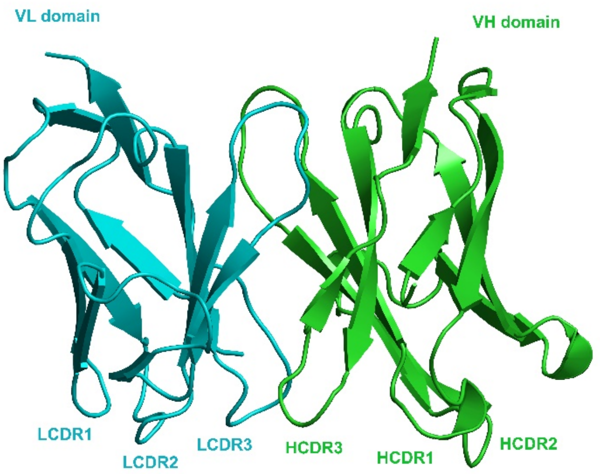

Light chain (VL) domain:

LCDR1 / LCDR2

AIQLTQSPSSLSASVGDRVTITC**RASQGISSALV**WYQQKPGKAPKLLIY**DASSLES**GVPSRFSGSG

+ (R); - (D), - (E)

LCDR3

SGTDFTLTISSLQPEDFATYYC**QQFNDYFTF**GPGTKVDIK

- (D)

Net charge of light chain: -2, and Net charge of LCDR3: -1

Heavy chain (VH) domain:

HCDR1 / HCDR2

QVQLVQSGAEVKKPGASVKVSCKASGYTFT**NHYMH**WVRQAPGQGLEWMG**IINPISGSTSNAQKFQG**RV

+ (H), + (H); + (K)

HCDR3

TMTRDTSTSTVYMELSSLRSEDTAVYYCAR**DIVDAFDF**WGQGTMVTVSS

- (D), - (D), - (D)

Net charge of heavy chain: 0, and Net charge of HCDR3: -3

Supplementary Figure S2. Homology model of Burosumab variable fragment (Fv). The structural model was generated using RosettaAntibody3 module from ROSIE webserver (https://rosie.rosettacommons.org/antibody/). The VL (residues 1-106) and VH (residues 1-117) domains with corresponding amino acid sequences are indicated in cyan and green, respectively. The bold letters indicate CDR residues that were selected for experimental alanine scanning. Positively charged and negatively charged CDR residues were denoted by + and -, respectively. Basic residues: Histidine (H), arginine (R), and lysine (K) were counted for charge as +1 per residue, while acidic residues: Aspartic (D) and glutamic (E) were counted for charge as -1 per residue. Structure was created by PyMOL version 2.2.0.

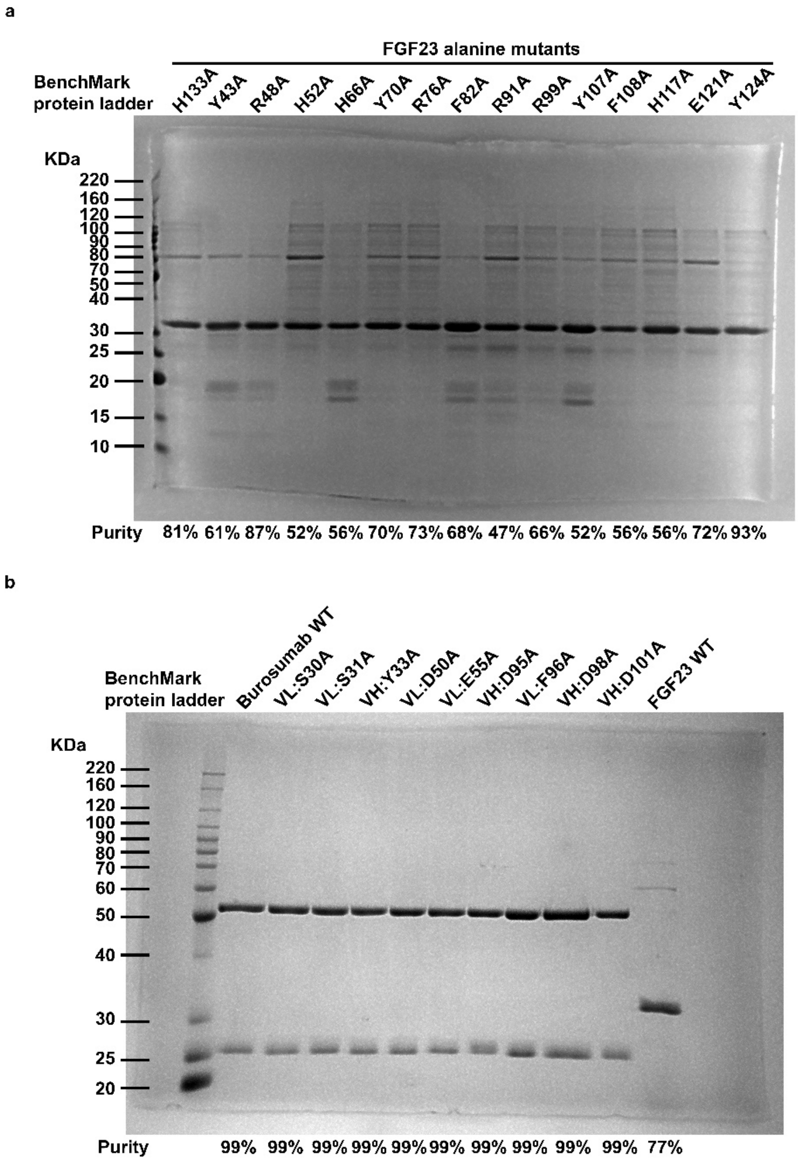

Supplementary Figure S3. SDS-PAGE analysis of (a) purified N-terminal domain of FGF23s having SUMO His tag and (b) Burosumab alanine variants. The purity of proteins was analyzed using SDS-PAGE gel containing 12% acrylamide under reducing conditions. Each mutant (1μg) was separated according to the molecular weight at a constant 200 voltages for 1 h. followed by Coomassie blue staining to visualize the protein purity. FGF23 alanine mutants migrated approximately at 32 KDa. Burosumab wild-type and mutants migrated approximately at 50 KDa (heavy chain) and 25 KDa (light chain). BenchMark protein ladder (Thermo Scientific) was used. The gel image was taken using ChemiDoc-It2 imager (Analytik Jena Company). The purity was determined using VisionWorks software.

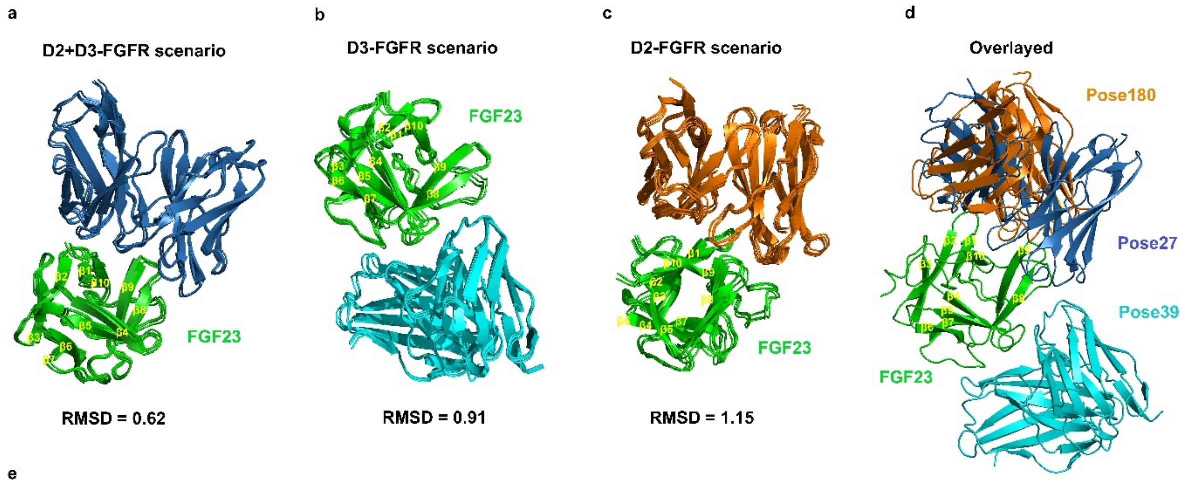

e

Epitope Restraint scenarios	Top ranked poses	Total energy (kcal/mol)	FGF23 blockage	Interface area (Å²)	Percentage framework residue contribution (%FWR)	Percentage CDR residue contribution (%CDR)	Percentage H3 and L3 residue contribution (%H3, %L3)	Percentage correlation with paratope alanine scanning	Rank in group
D2 + D3 FGFR	27	-339.61	strong	906.7	22.23	77.77	22.21, 21.27	75%	1
	156	-318.01	strong	928.5	20.40	79.60	18.57, 21.61	64%	3
	127	-371.35	strong	950.6	21.09	78.91	21.49, 18.63	69%	2
	182	-345.132	strong	912.9	19.74	80.26	20.96, 20.87	61%	4
D3 FGFR	39	-259.12	weak	859.3	13.47	86.53	15.91, 12.28	69%	1
	46	-179.94	weak	800.4	12.18	87.82	18.11, 27.63	64%	3
	47	-214.65	weak	776.7	12.08	87.92	18.59, 26.98	61%	4
	15	-204.39	weak	748.9	12.28	87.72	16.51, 26.03	67%	2
D2 FGFR	180	-306.53	strong	920.8	13.96	86.04	14.05, 26.9	61%	1
	177	-237.32	strong	973.1	18.37	81.63	12.83, 15.39	58%	2
	168	-276.38	strong	958.6	18.60	81.40	11.78, 18.58	58%	3
	140	-270.68	strong	937.2	17.61	82.39	13.41, 19.42	50%	4

Supplementary Figure S4. Comparison of top poses within and across epitope restraint scenarios and their assessment. HADDOCK2.4 was run using default parameters with the homology model of Burosumab Fv and the crystal structure of FGF23 (PDB ID: 5W21). Top-four ranked poses within each scenario: D2+D3 FGFR (a), D3 FGFR (b), and D2 FGFR scenarios (c) were superimposed to show their structural similarity. The Fv orientations from pose27, pose39 and pose180 were superimposed keeping FGF23 as the reference (d) to compare and contrast between these poses. Table (e) shows the structural metrics of these poses. Overall RMSD refers to root

mean square deviation between the atomic positions of superimposed poses. Superimposition of structures were performed using PyMOL version 2.2.0.

Supplementary Table S1. Top five lowest-energy Fv models of Burosumab obtained from ROSIE server. The Energy score was calculated based on Rosetta all-atom energy function which is a summation of individual energy terms (van der Waals, solvation, hydrogen bonding energies, etc.). SASA refers to solvent accessible surface area of the homology model. H1-3 refers to HCDR1-3, L1-3 refers to LCDR1-3, and whole Fv refers to combined VH and VL domains. The templates of canonical CDRs (H1-3, and L1-3) were grafted onto the selected framework templates (FRH and FRL), resulting in a set of crude starting structures. The template chosen for Fv was used to guide the VH:VL orientation. These initial structures were optimized to find the energetically favorable H3 loop orientations. The top five models were ranked based on the Energy score. The top ranked model (pose 224) was selected for docking with FGF23 (PDB: 5w21).

**Top 5 models**	**Energy score (kcal/mol)**	**CDR SASA (Å^2)**	**PDB templates used for CDR grafting and VH-VL orientation (% sequence identity with Burosumab)**								
			**FRH**	**FRL**	**H1**	**H2**	**H3**	**L1**	**L2**	**L3**	**whole Fv**
224	-301.28	3229	PDB: 3SQO (98%)	PDB: 1DFB (96%)	PDB: 2D7T (80%)	PDB: 2D7T (75%)	PDB: 1IQD (80%)	PDB: 1VGE (91%)	PDB: 3IDG (100%)	PDB: 3CMO (50%)	PDB: 3SQO (82%)
119	-299.35	3302									
148	-295.72	2990									
91	-286.27	3168									
50	-276.48	3255									

Supplementary Table S2. Summary of alanine scanning on VH domain of Burosumab using spot ELISA and/or indirect ELISA assays. Relative binding was calculated from spot ELISA of FGF23-Burosumab interaction by comparing an absorbance at 450 nm (A450) of each mutant with wild-type Burosumab (Methods). Fold decrease was calculated by comparing a K_d value of each mutant with wild-type Burosumab. CDR numbering was based on Chothia's definition.

#	Mutations	Binding assays	Average relative binding (%) from spot ELISA	K_d (nM) ± SD from indirect ELISA	Fold decrease in binding
	WT	spot ELISA/indirect ELISA	100	0.5 ± 0.03	nd
1	N31A	spot ELISA	119	nd	nd
2	H32A	spot ELISA	105	nd	nd
3	Y33A	spot ELISA/indirect ELISA	7	1389.11 ± 2180.46	>100-fold
4	M34A	spot ELISA	117	nd	nd
5	H35A	spot ELISA	67	nd	nd
6	I50A	spot ELISA	70	nd	nd
7	I51A	spot ELISA	105	nd	nd
8	N52A	spot ELISA	74	nd	nd
9	P52AA	spot ELISA	77	nd	nd
10	I53A	spot ELISA	87	nd	nd
11	S54A	spot ELISA	104	nd	nd
12	G55A	spot ELISA	97	nd	nd
13	S56A	spot ELISA	110	nd	nd
14	T57A	spot ELISA	119	nd	nd
15	S58A	spot ELISA	111	nd	nd
16	N59A	spot ELISA	107	nd	nd
17	A60A	spot ELISA	106	nd	nd
18	Q61A	spot ELISA	106	nd	nd
19	K62A	spot ELISA	111	nd	nd
20	F63A	spot ELISA	98	nd	nd
21	Q64A	spot ELISA	125	nd	nd
22	G65A	spot ELISA	124	nd	nd
23	D95A	spot ELISA/indirect ELISA	9	65.04 ± 12.4	>100-fold
24	I96A	spot ELISA	104	nd	nd
25	V97A	spot ELISA/indirect ELISA	119	nd	nd
26	D98A	spot ELISA/indirect ELISA	59	1.31 ± 0.03	2.62-fold
27	A99A	spot ELISA	104	nd	nd
28	F100A	spot ELISA	74	nd	nd
29	D101A	spot ELISA/indirect ELISA	51	0.69 ± 0.02	1.39-fold
30	F102A	spot ELISA	105	nd	nd

nd = not done.

Supplementary Table S3. Summary of alanine scanning on VL domain of Burosumab using spot ELISA and/or indirect ELISA assays. Relative binding was calculated from spot ELISA of FGF23-Burosumab interaction by comparing an absorbance at 450 nm (A450) of each mutant with wild-type Burosumab (Methods). Fold decrease was calculated by comparing a K_d value of each mutant with wild-type Burosumab. CDR numbering was based on Chothia's definition.

#	Mutations	Binding assays	Average relative binding (%) from spot ELISA	K_d (nM) ± SD from indirect ELISA	Fold decrease in binding
	WT	spot ELISA/indirect ELISA	100	0.5 ± 0.03	nd
1	R24A	spot ELISA	107	nd	nd
2	S26A	spot ELISA	89	nd	nd
3	Q27A	spot ELISA	86	nd	nd
4	G28A	spot ELISA	95	nd	nd
5	I29A	spot ELISA	68	nd	nd
6	S30A	spot ELISA/indirect ELISA	26	0.4 ± 0.06	0.8-fold
7	S31A	spot ELISA/indirect ELISA	52	0.37 ± 0.21	0.74-fold
8	L33A	spot ELISA	92	nd	nd
9	V34A	spot ELISA	108	nd	nd
10	D50A	spot ELISA/indirect ELISA	6	1099.47 ± 1871.08	>100-fold
11	S52A	spot ELISA	121	nd	nd
12	S53A	spot ELISA	129	nd	nd
13	L54A	spot ELISA	105	nd	nd
14	E55A	spot ELISA/indirect ELISA	50	0.51 ± 0.33	1.02-fold
15	S56A	spot ELISA	104	nd	nd
16	S67A	spot ELISA	69	nd	nd
17	T69A	spot ELISA	63	nd	nd
18	Q89A	spot ELISA	67	nd	nd
19	Q90A	spot ELISA	77	nd	nd
20	F91A	spot ELISA	52	nd	nd
21	N92A	spot ELISA	87	nd	nd
22	D93A	spot ELISA/indirect ELISA	48	0.36 ± 0.05	0.72-fold
23	Y94A	spot ELISA	57	nd	nd
24	F96A	spot ELISA/indirect ELISA	33	1.16 ± 0.12	2.32-fold
25	T97A	spot ELISA	103	nd	nd
26	F98A	spot ELISA	78	nd	nd

nd = not done.

Supplementary Table S4. Interacting residues of FGF23-Burosumab interaction based on the top-rank pose27, which was reported from BIOVIA Discovery Studio version 2017 R7 using a default setting.

Types of interaction	Interacting residues (FGF23 – Burosumab)	Distances (Å)
Hydrogen bond	Y43 [HH] --- VL:D50 [OD1]	1.91
Hydrogen bond	N122 [HD]--- VL:N92 [O]	2.06
Hydrogen bond	A47 [O]---VL:S30[HN]	2.34
Hydrogen bond	E163 [OE2]---VL:Y49[HH]	3.01
Hydrogen bond	Y124[OH]---VL:N92[HD21]	2.56
Hydrogen bond	R160 [HE]--- VH:D102 [OD2]	2.23
Hydrogen bond	L120 [O]--- VH:Y33 [HH]	2.28
Hydrogen bond	E121 [OE1]---VH:N52 [HD21]	1.66
Hydrogen bond	E121 [OE1]---VH:S55 [HG]	2.64
Attractive charge	R161[NH1]---VL:E55[OE1]	3.80
Attractive charge	R160[NH2]---VL:E55[OE1]	5.43
Pi-anion interaction	Y43---VL:D50[OD2]	4.01
Salt bridge; Attractive charge	R160 [HH22]---VH:D102[OD2]	2.54
Salt bridge; Attractive charge	R161[HH12]---VH:D102[OD2]	1.67
Salt bridge; Attractive charge	R161[HH22]---VH:D105[OD2]	1.93
